# Adsorption Capacity, Reaction Kinetics and Thermodynamic Studies on Ni(II) Removal with GO@Fe_3_O_4_@Pluronic-F68 Nanocomposite

**DOI:** 10.3390/polym17152141

**Published:** 2025-08-05

**Authors:** Ali Çiçekçi, Fatih Sevim, Melike Sevim, Erbil Kavcı

**Affiliations:** 1 Chemical Engineering Department, Atatürk University, Erzurum 25240, Türkiye; 2 Chemical Engineering Department, Kafkas University, Kars 36100, Türkiye; erbilkavci@gmail.com; 3 Chemistry Department, Scıence Faculty, Atatürk University, Erzurum 25240, Türkiye; melike.sevim@atauni.edu.tr; 4 Nanoscience and Nanoengineering Department, Atatürk University, Erzurum 25240, Türkiye

**Keywords:** nanocomposite, heavy metal removal, environmental remediation, wastewater treatment, adsorption capacity

## Abstract

In recent years, industrial wastewater discharge containing heavy metals has increased significantly and has adversely affected both human health and the aquatic ecosystem. The increasing demand for metals in industry has prompted researchers to focus on developing effective and economical methods for removal of these metals. In this study, the removal of Ni(II) from wastewater using the Graphene oxide@Fe_3_O_4_@Pluronic-F68 (GO@Fe_3_O_4_@Pluronic-F68) nano composite as an adsorbent was investigated. The nanocomposite was characterised using a series of analytical methods, including Fourier transform infrared spectroscopy (FT-IR), scanning electron microscopy (SEM), X-ray diffraction (XRD), and Brunauer-Emmett-Teller (BET) analysis. The effects of contact time, pH, adsorbent amount, and temperature parameters on adsorption were investigated. Various adsorption isotherm models were applied to interpret the equilibrium data in aqueous solutions; the compatibility of the Langmuir, Freundlich, Temkin, and Dubinin-Radushkevich models with experimental data was examined. For a kinetic model consistent with experimental data, pseudo-first-order, pseudo-second-order, Elovich, and intra-particle diffusion models were examined. The maximum adsorption capacity was calculated as 151.5 mg·g^−1^ in the Langmuir isotherm model. The most suitable isotherm and kinetic models were the Freundlich and pseudo-second-order kinetic models, respectively. These results demonstrate the potential of the GO@Fe_3_O_4_@Pluronic-F68 nanocomposite as an adsorbent offering a sustainable solution for Ni(II) removal.

## 1. Introduction

Advances in technology have been accompanied by an escalating degree of damage to the natural environment and human beings. In the current century, humanity is attempting to mitigate its deleterious impact. In the context of the evolving industry, the management of heavy metal waste released into the environment has emerged as a pressing concern. The uncontrolled release of heavy metals into water and soil has the potential to adversely impact all living organisms due to the toxic effects they can elicit. A future in which natural resources are diminishing on a daily basis and access to natural agricultural products and drinking water is becoming increasingly challenging awaits humanity. In this regard, nickel can be regarded as a potentially toxic heavy metal that poses a threat to the integrity of natural ecosystems. Nickel, a toxic heavy metal that is not biodegradable, can enter the human body through water and accumulate in tissues such as the lungs, intestines, and skin over time. A study of nickel refining workers revealed a high incidence of stomach and lung cancer, as well as severe bodily damage. Nickel, an element of increasing importance in numerous fields, including metal plating, mining, battery production, and paint industries, has garnered significant attention from the scientific community. A primary focus of researchers has been the prevention and elimination of nickel accumulation in water and soil ecosystems. One of the metal ions whose concentration in drinking water is restricted is Ni(II). According to the World Health Organization (WHO), the acceptable nickel level in drinking water is set at 0.02 milligrams per liter (mg·L^−1^). This is the maximum acceptable concentration for safe drinking water according to WHO [1].

Many methods are applied for the removal of nickel. The disadvantages of many separation methods, such as chemical precipitation, ion exchange, membrane separation, solvent extraction, and electrochemical treatment, high cost, and complex and long processes, have led science to search for alternatives. Adsorption is preferred alternative separation method because of its low cost, cost-effectiveness, and sustanable solution. In recent scientific research, it is noteworthy that the adsorption method is frequently preferred for the removal of impurities such as heavy metals and dyes. Mahdieh Namvar-Mahboub et al. chose adsorption, which is less costly and useful than many separation methods, such as chemical precipitation, ion exchange, membrane separation, solvent extraction, and electrochemical treatment, as a method of removing Pb and Cd from water [2]. Again, A. Muthukrishnaraj et al. preferred an adsorption process with Fe_3_O_4_ supported nanocomposite to remove Ni and Zn from water [3]. The adsorption process based on adsorption on the surface of the adsorbent used is directly related to the surface properties of the adsorbent and the adsorption mechanism. Therefore, new generation adsorbent materials providing higher capacity and selectivity are popular research topics [4].

With the advancement of nanotechnology, more functional materials are obtained thanks to frequently produced nanoparticles. Their main features are their small size and high surface area/volume ratio. Thanks to these properties, nanoparticles have become popular materials that adsorb heavy metals from water in recent period [4]. Graphene oxide, which has been frequently used in scientific research recently, is frequently used as a new generation adsorbent due to its large surface area, superior physical and chemical properties, and high adsorption capacity. In addition, the presence of functional hydroxyl, epoxy, and carboxyl groups containing a delocalized π-electron system and oxygen in the structure of graphene oxide makes it an ideal adsorbent for metal ions [5].

Nanoparticles with magnetic properties are frequently preferred in heavy metal adsorption due to their selective magnetic properties and electromagnet [6]. Magnetite (Fe_3_O_4_) with oxidative stability, which we benefit from its magnetic properties, is a frequently preferred nanoparticle to form non-toxic magnetic materials for adsorption applications. Magnetic nanoparticles composed of materials such as cobalt, nickel, and neodymium, iron, boron can offer improved magnetic properties. However, these materials are dangerous for human health due to their susceptibility to oxidation. Surfactants, which significantly change the surface and interfacial properties of the substances they are incorporated into, are frequently used in new-generation adsorbents. Nonionic surfactants do not contain anionic (negatively charged) or cationic (positively charged) functional groups. Thus, charge stability is maintained on the surface. Thanks to their amphiphilic properties, nonionic surfactants can undertake functions such as reducing surface tension, forming emulsions, and providing stabilization in systems with changing pH [7].

In this study, GO-Fe_3_O_4_ nanocomposites were synthesized by the liquid-phase self-assembly method, graphene oxide was synthesized by the modified Hummers method [8], and Fe_3_O_4_ nanoparticles were synthesized [9]. Pluronic F-68, which has nonionic properties, was used to eliminate the variable state of Fe_3_O_4_. Here, it took advantage of its supermagnetic properties against environmental conditions [10,11]. Pluronic F-68 is a type of surfactant and is a copolymer consisting of hydrophilic and hydrophobic blocks. Due to these properties, Pluronic F-68 increases the dispersion stability of the materials that are used in the modification of the surface [3,12]. Thus, we have modified the GO-Fe_3_O_4_ nanocomposite with Pluronic F-68 polymer and then utilized this nanocomposite for Ni(II) removal from wastewater. We believe that using the synthesized GO- Fe_3_O_4_-Pluronic F68 nanocomposite to remove heavy metal (Ni(II) will add innovation to the literature.

In this study, the fundamental characteristics of the adsorption process used in the removal of heavy metal ions were elucidated by analyzing experimental data with four different kinetic models. The kinetic models quantitatively evaluated the adsorption rate and time-dependent behavior, allowing the dominant mechanism (physical/chemical) of the process to be determined.

Four different models were discussed to determine the Ni^2+^ adsorption capacity and surface properties under equilibrium conditions. These models revealed the maximum metal ion retention capacity (qmax) of the adsorbent, whether adsorption occurs on homogeneous/heterogeneous surfaces, and the physical/chemical nature of the process.

Thermodynamic parameters (ΔG°, ΔH°, ΔS°) were calculated to assess the energetic feasibility of the process. Gibbs free energy (ΔG°) values revealed the degree of spontaneity of adsorption, while enthalpy (ΔH°) changes determined the endothermic/exothermic character of the process. Entropy (ΔS°) values quantified the change in disorder in the system. These findings provide a detailed explanation of the relationship between the process and temperature and its practical applicability.

The study also included optimization studies conducted under different pH, temperature, and initial metal ion concentrations, supported by statistical analyses of model compatibility. This comprehensive approach demonstrated the modelability of the developed adsorption system and demonstrated the process’s robustness against operational variables encountered in industrial applications.

## 2. Experimental Details

### 2.1. Materials

The following reagents were utilized: iron(III) acetylacetonate (Fe(acac)_3_, 97%), oleic acid (OAc, 90%), oleylamine (OAm, ≥70%), benzyl ether (BE, 99%), potassium permanganate (KMnO_4_, ≥99%), and sodium nitrate (NaNO_3_) ≥ 99.0%). The following substances were obtained from Sigma-Aldrich, Germany: dimethylformamide (DMF, ≥99%), hexane (97%), isopropanol (99%), ethanol (99%), acetone (97%), and NiCI_2_(H_2_O)_6_ (97%).

The hydrogen peroxide (H_2_O_2_, 30%) and sulfuric acid (H_2_SO_4_, 95–98%) utilized in this study were obtained from Merck, Germany. Natural graphite sheets were procured from Alfa-Aesar, Haverhill, MA, USA. Nonionic surfactant was obtained in liquid form (10%) from Thermo Fisher Scientific, Waltham, MA, USA.

### 2.2. Synthesis of GO@Fe_3_O_4_@Pluronic F-68 Nonacomposite

Fe_3_O_4_ nanoparticles were synthesised according to the method described in the literature [13]. As the first step of the synthesis, 1.06 g of Fe(acac)_3_, 15 mL of oleylamine, and 15 mL of benzyl ether were mixed at room temperature under a nitrogen flow. The mixture was heated to 120 °C under a 500 rpm stirrer, and the reaction temperature was maintained at this temperature for 1 h. The reaction temperature was then increased to 285 °C and maintained at this temperature for 1 h. The resulting Fe_3_O_4_ was dissolved in hexane and transferred to centrifuge tubes. The hexane-dissolved sample was centrifuged twice with the addition of ethanol. Fe_3_O_4_ nanoparticles were obtained and stored in hexane.

The 400 mg of graphene oxide obtained using the modified Hummer’s method was dispersed in 40 mL of DMF using a sonicator for approximately 15 min. The Fe_3_O_4_ nanoparticles stored in hexane were added dropwise to this graphene oxide solution, and sonication was applied for 2 h [14]. As a result we obtained Fe_3_O_4_-GO solution. Subsequently, Pluronic F68 does not contain anionic (negatively charged) or cationic (positively charged) functional groups. Thus, charge stability is maintained on the surface. Thanks to their amphiphilic properties, nonionic surfactants can undertake functions such as reducing surface tension, forming emulsions, and providing stabilization in systems with changing pH. Pluronic F-68 is a nonionic triblock copolymer characterized by a central hydrophobic poly(propylene oxide) (PPO) segment and two hydrophilic poly(ethylene oxide) (PEO) segments. The PPO block typically contains 25–30 repeat units, while each PEO block consists of approximately 75–85 ethylene oxide (EO) units on average. This amphiphilic structure confers surfactant properties to Pluronic F-68, enabling it to exhibit both surface-active behavior and polymeric functionality [15]. Pluronic F-68′s nonionic structure makes the resulting composite more stable against pH changes 1 mL of Pluronic F-68 was added by dropwise to Fe_3_O_4_-GO solution and dispersed for 2 h using a sonicator. The solution was then treated with ethanol to remove DMF and centrifuged at 8500 rpm for 10 min. The resulting solid material was dried at 70 °C for 12 h. The dried nanocomposite material (GO@Fe_3_O_4_@Pluronic-F68) was obtained. The nanocomposite was used for characterisation and absorption experiments.

### 2.3. Characterisation

Fourier transform infrared (FT-IR) analyses were performed to evaluate the chemical bond formations of the obtained GO@Fe_3_O_4_@Pluronic-F68 (Bruker VERTEX 70v). X-ray diffraction (XRD) patterns was studied on a PANalytical Empyrean diffractometer with Cu-Kα radiation (40 kV, 15 mA, 1.54051 Å) over a 2θ range from 20−80° at room temperature. SEM analyses were performed with the FEI INSPECT550 device. Surface analysis was performed by nitrogen adsorption and desorption on BET Micromeritics 3 Flex. Thermo ICAP TQ brand ICP-MS device was used for Ni(II) analyses.

### 2.4. Adsorption Experiments

To investigate the nickel adsorption of the GO@Fe_3_O_4_@Pluronic-F68 material we obtained, NiCI_2_(H_2_O)_6_ 97% purity solid was dissolved in pure water, and a stock solution containing 1000 mg·L^−1^ Ni (II) ion was prepared. The solutions used in the experiments were prepared from the stock solution. Samples of 50 mL were taken from the determined solutions, and experiments were carried out on a shaker at 185 rpm by adding 100 mg GO@Fe_3_O_4_@Pluronic-F68 material. The batch experiments were carried out at 5–10–20–30–60–120 min. At the end of the time, the samples taken from each experiment were centrifuged at 9000 rpm to separate the adsorbent residues. ICP-MS was then used to determine the Ni(II) concentration in the filtrate. The variation of pH between 2–8 was analyzed for Ni(II) adsorption. To investigate the effect of temperature on adsorption capacity, four different temperatures (20–25–30–35 °C) were studied. Initial concentration values were taken as 100–200–300–400 mg·L^−1^. The adsorbent amount was studied at 25–50–75–100–150 mg values.

The amount of metal ion adsorbed on the adsorbent at equilibrium, q_e_, was calculated with the help of Equation (1):

(1) $$q_{e}=(C_{0}-C_{e})\frac{V}{m}$$

Here, V: volume of the solution (L), C_o_: initial concentration of the solution (mg·L^−1^), C_e_: adsorbate concentration remaining in the solution after adsorption (mg·L^−1^), m adsorbent amount (g), q_0_: the amount of adsorbate adsorbed on unit adsorbent before adsorption (mg·g^−1^) [16].

### 2.5. Adsorption Isotherms

The development of mathematical models requires the analysis of equilibrium data obtained from adsorption experiments and Ni(II) removal. Adsorption isotherms are utilized to accurately describe adsorption for design purposes. The most commonly used isotherm models for aqueous solutions are Langmuir, Freundlich, Temkin and Dubinin-Radushkevich isotherm models [17].

### 2.6. Langmuir Isotherm

Langmuir isotherm is a system of equations, all with equal energy, designed for specific areas on the adsorbent surface and reversible adsorption. The linearised expression of Langmuir adsorption isotherm:

(2) $$\frac{C_{e}\;}{q_{e}}=\frac{1}{Q\cdot K_{L}}+\frac{C_{e}}{Q}$$

q_e_ = Amount of adsorbed substance per unit adsorbent weight, (mg·g^−1^)
Q = Adsorption capacity (mg·g^−1^)
K_L_ = Energy related constant (L·mg^−1^)
C_e_ = Concentration of substance remaining in solution after adsorption (mg·L^−1^)

is called the Langmuir isotherm equation. When C_e_/q en is plotted against C_e_, a linear line will be obtained [18].

### 2.7. Freundlich Isotherm

Freundlich isotherm reveals the relationship between the amount adsorbed at a certain concentration level and the concentration.

The mathematical expression of Freundlich isotherm is as follows;

(3) $$In\;q_{e}=In\;K_{F}+(1/n)In\;C_{e}$$

Here:

q_e_ = Amount of adsorbed substance on a unit adsorbent (mg·g^−1^)

K_F_ = Temperature dependent Freundlich adsorption capacity constant [(mg·g^−1^) (L·g ^−1^)^−1/n^]

n = A constant representing the temperature dependent adsorption intensity

C_e_ = The concentration of adsorbate remaining in solution at equilibrium (mg·L^−1^). The constants K_F_ and n are found by graphing the change of log q_e_ versus log C_e_ [19].

### 2.8. The Temkin Isotherm

The Temkin isotherm is used to best explain adsorbate-adsorbent interactions and binding energies. It is expressed in Equation (4) [19].

(4) $$q_{e}=B_{T}\times \ln\left(K_{T}\right)+B_{T}\times \ln\left(C_{e}\right)$$

where $B_{T}=RT/b_{T}$.

### 2.9. The Dubinin–Radushkevich Isotherm

The Dubinin–Radushkevich isotherm is used to estimate the porosity of the adsorbent, the Gaussian energy distribution of a heterogeneous surface and apparent adsorption energies (especially in porous adsorbents). This model equation is given in Equation (5) [19]

(5) $$ln\left(q_{e}\right)=\ln\left(q_{m}\right)-\beta \;\times \;\varepsilon^{2}$$

where ε, is the Polanyi potential, $\varepsilon =R_{g}\mathrm{Tln}\left(1+\frac{1}{C_{e}}\right)$.

### 2.10. Kinetic Studies

Kinetic models in adsorption allow us to obtain information about the adsorption rate, the modeling of the process, and whether the adsorbent/adsorbate interaction is physical or chemical [20].

### 2.11. Pseudo-First Order Reaction Kinetics Model

The pseudo-first-order kinetic model is expressed as follows (6).

(6) $$\log\left(q_{e}-q_{t}\right)=logq_{e}-\frac{k_{1}t}{2.303}\;$$

where q_e_ is the amount of adsorbed substance at equilibrium (mg·g^−1^), q_t_ is the amount of adsorbed substance at any time t (mg·g^−1^), and k_1_ (min^−1^) is the adsorption rate constant [20].

### 2.12. Pseudo-Second Order Reaction Kinetics Model

The pseudo-second-order kinetic model is expressed as follows (7).

(7) $$\frac{t}{q_{t}}=\frac{1}{k_{2}q_{e}^{2}}+\frac{1}{q_{e}}t\;$$

where k_2_ is the adsorption rate constant (g·mg^−1^·min^−1^), q_e_ is the amount of adsorbed substance at equilibrium (mg·g^−1^), and it is the amount of adsorbed substance at any time t (mg·g^−1^) [21].

### 2.13. Elovich Reaction Kinetics Model

The Elovich kinetic model, developed to explain the chemical adsorption of gases on heterogeneous solid surfaces, is given below (8):

(8) $$q_{t}=\frac{ln(\alpha \beta )}{\beta }+\frac{lnt}{\beta }$$

In the equation, α represents the initial sorption rate (mg·g^−1^·min^−1^), and β represents the surface activation energy (mg·g^−1^) required for chemical sorption. q_t_: The amount of adsorbate at time t (mg·g^−1^) [22].

### 2.14. Intraparticle Diffusion Reaction Kinetics Model

The intraparticle diffusion model is expressed as follows (9).

(9) $$q_{t}=K_{i}\sqrt{t}+C\;$$

Here:

K_i_ = intraparticle diffusion rate constant (mg·g^−1^·min^−2^)

C = a constant that gives information about the thickness of the layer formed between adsorbent and adsorbate The

C value is calculated from the intercept point of the graph of the rate constant K_i_, q_t_ versus $\;\sqrt{t}$ [23].

### 2.15. Thermodynamic Studies

Gibbs free energy change (∆G°), enthalpy change (∆H°) and entropy change (∆S°), and thermodynamic analysis of the adsorption process were calculated using the following equations (Equations (10)–(12)) [17].

(10) $$K_{c}=\frac{q_{e}}{C_{e}}$$

(11) $$ln(K_{c})=(∆S{}^{\circ}/R)-(∆H{}^{\circ}/RT)$$

(12) $$∆G{}^{\circ}=-RT\times \ln\left(K_{c}\right)=∆H{}^{\circ}-T∆S{}^{\circ}$$

Here:

ΔG°: Free energy change (kJ·mol^−1^)

ΔH°: Enthalpy change (kJ·mol^−1^)

ΔS°: Entropy change (kJ·mol^−1^·K)

T: Absolute temperature (K)

R: Gas constant (8.314 J·mol^−1^·K)

K_C_: Equilibrium constant

## 3. Results and Discussion

### 3.1. Characterisation

**Figure 1:**
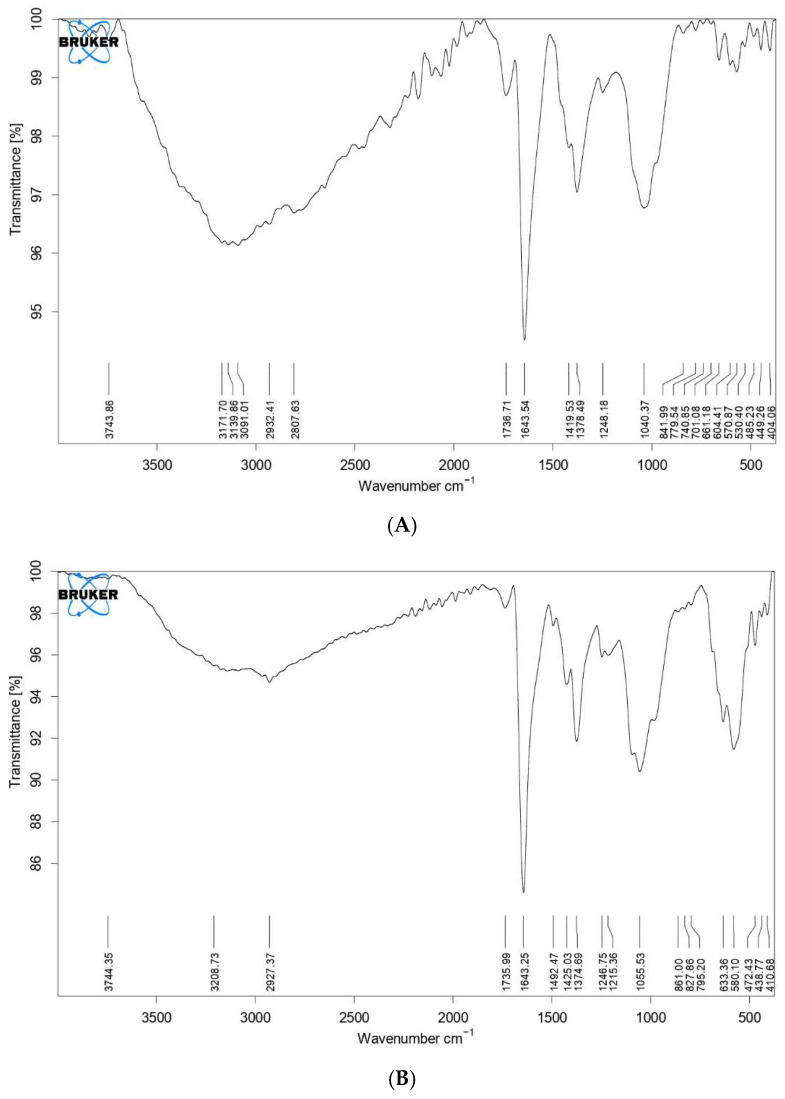
FT-IR Spectrum Analysis Results (**A**) GF FT-IR spectrum, (**B**) GO@Fe_3_O_4_@Pluronic-F68 FT-IR spectrum.

The vibrations around 570 and 661 cm^−1^ in Figure 1A were attributed to (Fe-O) bonds from iron oxide. The stretching vibrations at 1040 cm^−1^ and 1248 cm^−1^ indicate an epoxy bond (C-O) and alkoxy bond (C-O), respectively [24]. Vibrations around 1378 cm^−1^ indicated that a carboxyl (O=C-O) complex bond was obtained [25]. Vibrations occurring at 1643 cm^−1^ refer to aromatic (C=C) bonds defined as skeletal bonds of graphene. 1736 cm^−1^ indicated the presence of a carbonyl bond (C=O), while the broad diffuse adsorption band at 3743 cm^−1^ indicated the presence of a hydroxyl (O-H) bond [26]. The presence of epoxy and alkoxy bonds indicated that graphene was successfully oxidized. The results obtained from FT-IR spectroscopy showed that graphene oxide-Fe_3_O_4_ nanocomposite material was obtained.

Figure 1B shows the approximate values of the characteristic peaks of Pluronic^®^ F68 obtained in the studies of N Rarokar et al. in FTIR spectroscopy at 1215, 1246, 1425, (cm^−1^) [26]. Vibrations around 1055 cm^−1^ proved the presence of the characteristic absorption band of ether (C-O-C) in the structure of the Pluronic F-68 surfactant. The presence of alkane (C-H) and hydroxyl (O-H) bonds in the structure of Pluronic F-68 surfactant was proved by vibrations around 2927 cm^−1^ and 3208 cm^−1^, respectively [27]. The peak at 403 cm^−1^ in Figure S1 was attributed to Ni-N bonding, while the peak at 519 cm^−1^ confirmed Ni-O bonding (Figure 1). The FT-IR results obtained proved that Ni(II) adsorption occurred.

**Figure 2:**
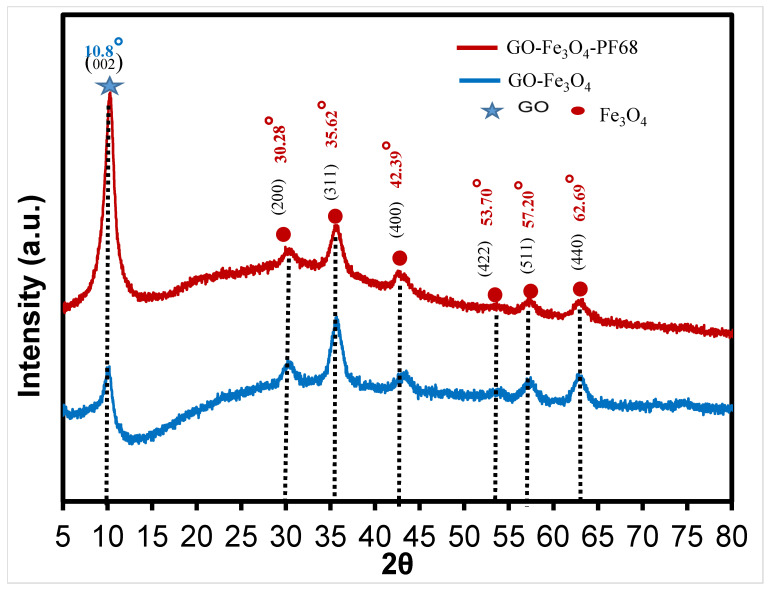
XRD patterns of Graphene Oxide-Fe_3_O_4_, GO@Fe_3_O_4_@Pluronic-F68 nano composite.

The X-ray diffraction pattern of graphene oxide-Fe_3_O_4_ material is presented in Figure 2, the prominent peak observed at 2θ = 10.8° represents the diffraction signal of the characteristic (002) plane of graphene oxide and confirms the presence of graphene oxide in the structure of the material. XRD analysis results reveal the presence of a spinel cubic structure in magnetic Fe_3_O_4_, as evidenced by the 2θ values of 30.28°, 35.62°, 42.39°, 53.70°, 57.20°, and 62.69°. The diffraction surfaces of the six characteristic peaks obtained were determined as ((220), (311), (400), (422), (511) and (440)) and the strongest peak was observed at 2θ = 35.68° in the XRD pattern [24]. In light of the literature research, these peaks belong to magnetic Fe_3_O_4_ and graphene oxide, the diffraction pattern is compatible with the literature [28]. The approximate values of the characteristic diffraction peaks of Pluronic F-68 obtained by N. Rarokar et al. were 22.7 and 25.76 in the XRD pattern [26].

Figure 2 shows the XRD pattern of the ternary complex material formed by binding the Pluronic F-68 surface-activating agent to graphene oxide and the Fe_3_O_4_ nanocomposite material. Following modification, it was observed that the Fe metal was bound to the graphene oxide, with the crystal structure remaining intact [29]. It was found that the XRD values obtained by binding the Pluronic F-68 surface activating agent caused partial shifts (Figure 2).

**Figure 3:**
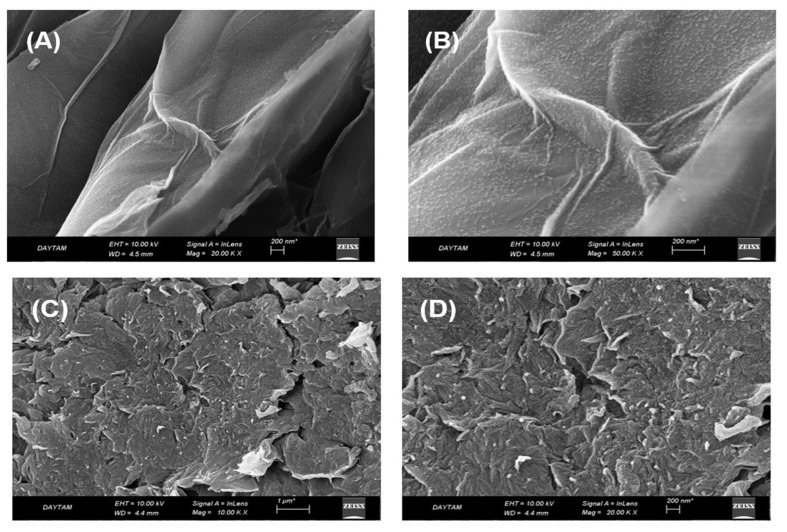
SEM images of (**A**,**B**) GO-Fe_3_O_4_ structure (**C**,**D**) GO-Fe_3_O_4_-PF-68 structure.

In Figure 3A,B, images of the GO-Fe_3_O_4_ structure were taken at different scales before loading the polymeric material. In both images, the paper structure of graphene oxide was seen, Fe_3_O_4_ nanoparticles were dispersed homogeneously onto the graphene oxide surface. Figure 3C,D show SEM images of the GO@Fe_3_O_4_@Pluronic-F68 structure. The binding of the polymeric structure is evident from the transformation from an elongated and curved shape in the image to a porous and dispersed shape.

**Figure 4:**
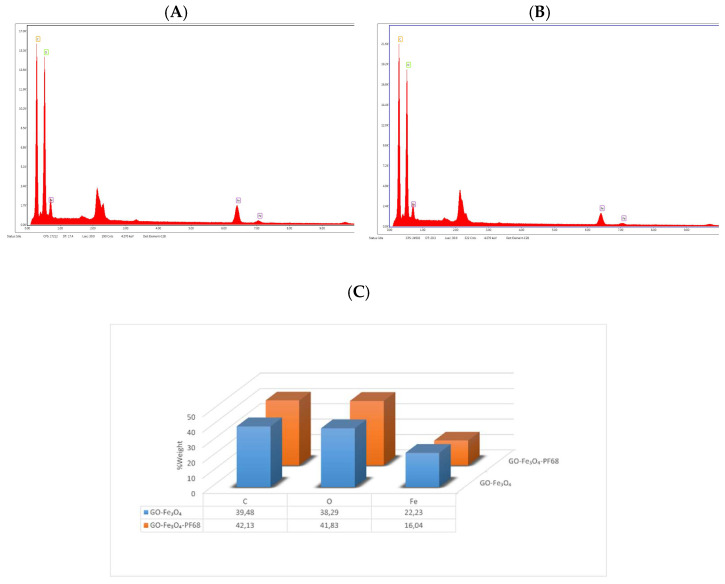
EDX analysis of (**A**) GO-Fe_3_O_4_ (**B**) GO@Fe_3_O_4_@Pluronic-F68 structure and (**C**) % weights of GO-Fe_3_O_4_, GO@Fe_3_O_4_@Pluronic-F68.

It was observed that the percentage of carbon and oxygen by weight increased in the complex material with the incorporation of Pluronic^®^ F68, containing dense C and O atoms into the magnetite graphene complex (Figure 4). This proved that Pluronic^®^ F68 was successfully incorporated into the structure of the adsorbent obtained.

**Figure 5:**
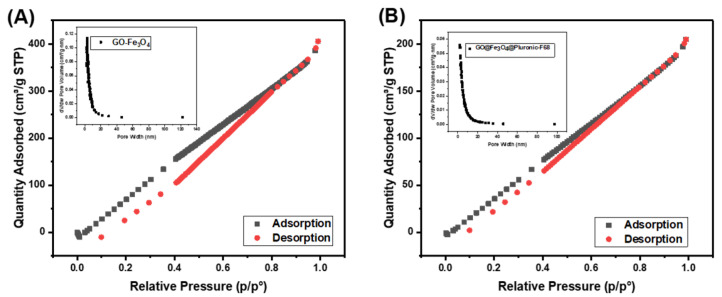
BET surface analysis of (**A**) GO-Fe_3_O_4_ and (**B**) GO@Fe_3_O_4_@Pluronic-F68.

After that, BET analysis was made for analysis to determine surface area measurements, micro, meso, and macro pore size, and pore size distribution by physical adsorption method in solid or powder samples at low pressures and high resolution (Figure 5). BET analysis determined that GO-Fe_3_O_4_ corresponded to a surface area of 459.2 m^2^. After modification with PF-68 surfactant, it was found to have a surface area of 236.2 m^2^·g^−1^. These results proved that PF-68 was incorporated into the GO-Fe_3_O_4_ surface.

The decrease in surface area observed in the BET analysis indicates that the Pluronic F-68 polymer successfully adsorbed onto the material surface or into its pores. Pluronic F-68 partially filled the micro/mesopores, reducing the accessible surface area. However, Pluronic F-68 can increase its selectivity toward certain molecules by modifying its surface chemistry, thanks to its hydrophilic PEO (polyethylene oxide) and hydrophobic PPO (polypropylene oxide) chain structure [30,31,32].

Pluronic F-68 (PF-68), a triblock copolymer (PEO-PPO-PEO), plays a versatile role in adsorbent systems. First, it increases colloidal stability by stabilizing the surface charge. Second, as a surfactant, it reduces surface tension, enabling the adsorbent to disperse homogeneously in aqueous solutions. The third and most critical function is to form coordination bonds with heavy metal ions (Pb^+2^, Cd^+2^, Cu^+2^ and Ni^+2^) via ether oxygens (-CH_2_-O-CH_2_-) in poly(ethylene oxide) (PEO) chains [15,33]. The Ni-O bond in the FT-IR analysis supports this (Figure S1). While the maximum Ni(II) adsorption capacity obtained with GO/Fe_3_O_4_ adsorbent by A. Muthukrishnaraj et al. was previously 121.5 mg g^−1^, in our study the maximum capacity increased to 151.5 mg g^−1^ [3]. This increase may be due to the functional groups in the surfactant. Similarly, the maximum adsorption capacity for Ni(II) adsorption by GO/Fe_3_O adsorbent was found to be 15.5 mg g^−1^ by Hua-Wei Chen et al. Layered carbon structures such as graphene and graphene oxide (GO) exhibit π-π interactions, which play a significant role in heavy metal adsorption. These interactions occur primarily through the overlap of electron clouds between aromatic rings and increase adsorption capacity. The addition of magnetic Fe_3_O_4_ nanoparticles facilitates the retention of cationic metal ions (Pb^+2^, Cd^+2^, Cu^+2^, and Ni^+2^) through electrostatic attraction [34]. This result reveals that in addition to the π–π interaction and electrostatic attraction obtained with the GO/Fe_3_O_4_ adsorbent, the functional groups ((poly(ethylene oxide) (PEO) chains) present in pluronic f-68 are beneficial to the adsorption.

### 3.2. Batch Adsorption Experiments

#### 3.2.1. Effect of pH

**Figure 6:**
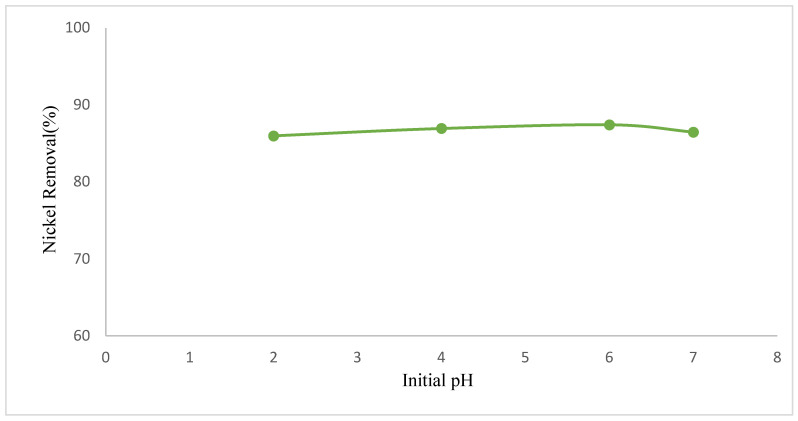
Effect of initial pH of the adsorption on GO@Fe_3_O_4_@Pluronic-F68 (C_0_: 200 mg·L^−1^, T: 20 °C, m: 100 mg, 185 rpm).

Since nickel hydroxide precipitates in the form of nickel hydroxide at pH values greater than 8, pH experiments for adsorption of Ni(II) were performed at initial pH values below 8 (pH = 2, 4, 6, 7). As can be seen from Figure 6, about 87% of nickel was adsorbed at all pH values, although the initial pH varied from 2 to 7. Since an adsorption mechanism that was not affected much by pH change emerged, the next experiments were performed at free pH (pH = 7). This may be because the nonionic surfactant Pluronic F-68, which we used during the synthesis of the complexing agent, causes ionic stability on the surface and provides stabilization against the changing pH of the medium [35,36].

It was also confirmed that complex forms such as Ni(OH)^+^ Ni(OH)^+2^ were formed in the solution at pH ≥ 8, but no accurate data could be obtained at pH 10. If the metal ion starts to precipitate in a certain pH range, the adsorption process is not directly affected by pH. Because metal ions are already precipitated out of the solution. Önal et al. investigated the removal of Ni(II) ions in the range of pH 2–10 with activated waste Malatya apricot, and it was determined that Ni(OH)^+2^ complex was present at pH ≥ 8 [37,38].

Nickel hydroxide (Ni(OH)_2_) is a sparingly soluble compound that dissociates in water according to the following equilibrium:

Ni(OH)_2(s)_ ⇌ Ni^+2^_(aq)_ + 2 OH^−^_(aq)_

$$[{\mathrm{Ni}}^{+2}]\min=\frac{K_{sp}}{{\left[{OH}^{-}\right]}^{2}}$$

The solubility product constant (K_sp_) for this reaction is given by:

For precipitation to occur, the ion product (Q) must exceed K_sp_.

At pH > 7, the solution becomes basic, and the hydroxide ion concentration [OH^−^] increases.

According to Le Chatelier’s Principle, higher [OH^−^] shifts the equilibrium leftward, promoting Ni(OH)_2_ precipitation.

The minimum [Ni^+2^] required for precipitation decreases as pH increases:

Thus, higher pH facilitates Ni(OH)_2_ precipitation because less free Ni^+2^ is needed to exceed K_sp_.

Conclusion; Ni(OH)_2_ precipitates at pH > 7 because, OH^−^ ions increase and this new situation cause the equilibrium shifts toward to the precipitation. The solubility of Ni^+2^ ions decreases. At high pH, hydroxo complexes become unstable and solidify [39,40].

#### 3.2.2. Effect of Contact Time and Initial Concentration

**Figure 7:**
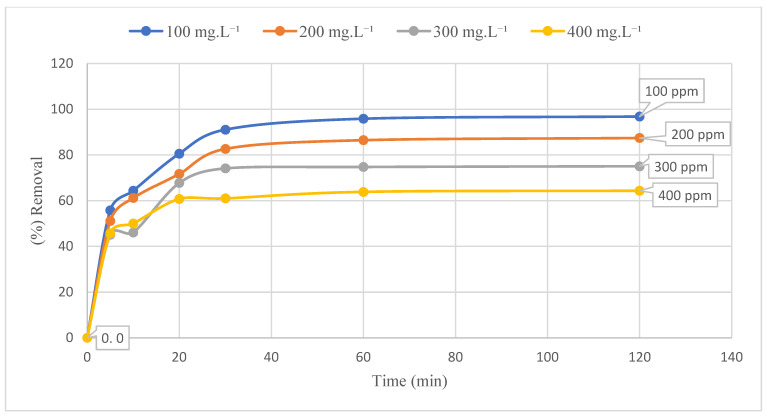
Effect of contact time and initial concentration (T: 20 °C, pH: 7.0, C_0_: 200 mg·L^−1^, 185 rpm, adsorbent dosage: 0.1 g).

As demonstrated in Figure 7, the effect of contact time and initial concentration of Ni(II) on the adsorption of Ni(II) on GO@Fe_3_O_4_@Pluronic-F68 is illustrated. Contact time was analyzed from 0 to 120 min, and initial concentration was measured from 100 milligrams per liter (mg·L^−1^) to 400 mg/L. In the course of the experiments, Ni(II) adsorption occurred expeditiously during the initial 30 min. Thereafter, a decline in the rate of adsorption was observed until the 60th minute, after which no further alterations were detected in the subsequent minutes.

After the 60th minute, negligible changes were observed, so 60 min was determined as the equilibrium time. 95.85% of 100 mg·L^−1^ Ni(II) solution, 84.47% for 200 mg·L^−1^, 74.74% for 300 mg·L^−1^ and 63.86% for 400 mg·L^−1^ were adsorbed. An increase in the initial nickel concentrations from 100 mg·L^−1^ to 400 mg·L^−1^ resulted in an enhancement of the adsorption capacity at equilibrium from 48 mg·g^−1^ to 129 mg·g^−1^ for GO@Fe_3_O_4_@Pluronic-F68. Concurrently, the adsorption percentage exhibited a decline from 95.85 to 63.86. As illustrated in Figure 7, an increase in the concentration of Ni(II) ions was observed, concurrent with a decrease in the percentage of adsorbed Ni(II). At the same time, the amount of adsorbed Ni increased with increasing concentration. Here, the driving force effective in mass transfer increased with increasing concentration, and therefore it was easier to overcome the mass transfer resistance between the aqueous phase and GO@Fe_3_O_4_@Pluronic-F68. Thus, the amount of adsorbed Ni(II) increased [41,42].

#### 3.2.3. Effect of Adsorbent Amount

**Figure 8:**
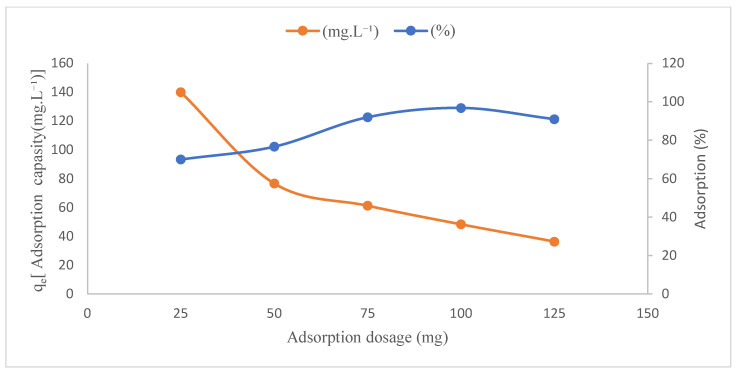
Effect of adsorbent dosage (C_0_: 200 mg·L^−1^, T: 20 °C, pH: 7.0, 185 rpm, t: 60 min).

Figure 8 shows the effect of the adsorbent amount. 25–50–75–100–150 mg GO@Fe_3_O_4_@Pluronic-F68 adsorbent was added to 100 mg.L^−1^ Ni(II) solution. Using 25 mg of adsorbent, 66.25% of Ni(II) was removed, while for 150 mg GO@Fe_3_O_4_@Pluronic-F68, 98.72% of Ni(II) was adsorbed. While the amount of material increased, the adsorption capacity decreased from 133 mg·g^−1^ to 33 mg·g^−1^. Increasing the amount of adsorbent usually increases the adsorption percentage. The reason for this is that the number of active sites increases with increasing adsorbent amount [43,44].

#### 3.2.4. Effect of Temperature

**Figure 9:**
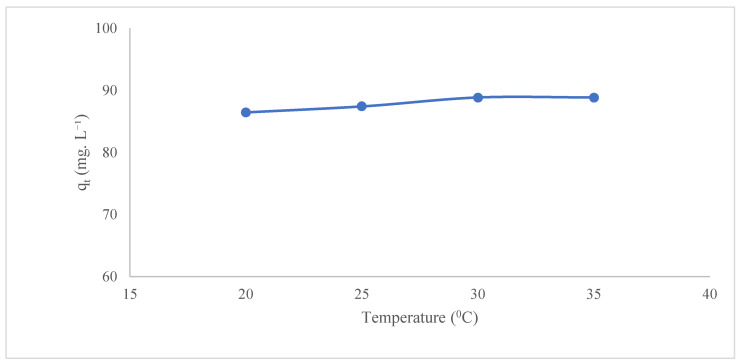
Effect of temperature (185 rpm, C_0_:200 mg·L^−1^, adsorbent dosage: 0.1 g, t: 60 min, pH: 7).

As illustrated in Figure 9, the impact of temperature on the adsorption of Ni(II) onto GO@Fe_3_O_4_@Pluronic-F68 is demonstrated. The experimental results indicated that 86.47% of Ni(II) was adsorbed at 20 °C, while 88.86% was adsorbed at 35 °C. An increase in temperature from 30 °C to 35 °C was observed, yet no significant change was detected. The adsorption capacity was calculated as 86 mg·g^−1^ at 20 °C and 89 mg·g^−1^ at 35 °C. The increase in temperature helps the kinetic energy of the molecules to increase and the metal ions to diffuse more quickly and effectively to the adsorbent surface. The reason for the partial increase observed in adsorption was the increase in the diffusion rate to the adsorbent surface due to temperature [45,46].

### 3.3. Adsorption Isotherm Studies

**Figure 10:**
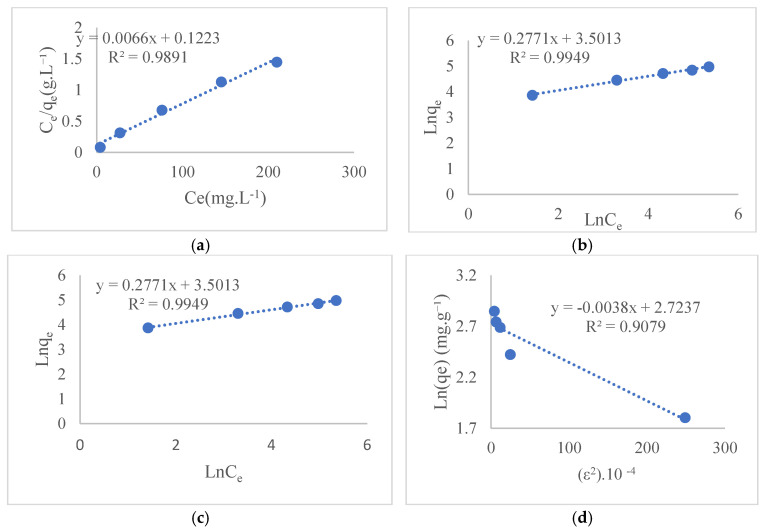
Isotherm curves for different isotherm models (**a**)—Langmuir, (**b**)—Freundlich, (**c**)—Temkin, and (**d**)—Dubinin-Radushkevich (T: 20 °C pH: 7.0 adsorbent dosage: 0.1 g t: 60 min).

The data obtained during the adsorption process of Ni(II) on GO@Fe_3_O_4_@Pluronic-F68 were investigated for compatibility with Langmuir, Freundlich, Temkin, and Dubinin-Radushkevich isotherm models. Figure 10 shows the GO@Fe_3_O_4_@Pluronic-F68 isotherms of Ni(II) adsorption. Table 1 shows the constants and correlation coefficients of the isotherms.

The Langmuir isotherm provides a model that assumes that adsorption occurs in a single layer and that the adsorption sites have the same energy. The maximum adsorption capacity (qm) seen in a single layer for GO@Fe_3_O_4_@Pluronic-F68 was calculated as 151.5 mg·g^−1^ and the correlation coefficient as 0.989. The Langmuir isotherm dimensionless separation factor (R_L_) values for the adsorbent at an initial concentration of 100–500 ppm (mg·L^−1^) are given in Table 1.

It is seen from Table 1 that the R_L_ values are between 0 and 1. When the R_L_ value is 0 < R_L_ < 1, adsorption is favorable; when R_L_ = 0, the system is irreversible; and when R_L_ > 1, adsorption is unfavorable [47]. Therefore, the adsorption of Ni(II) on GO@Fe_3_O_4_@Pluronic-F68 is favorable. Freundlich isotherm presents an adsorption model in which multilayer adsorption is observed as defined for heterogeneous systems. In Table 1, it is seen that the correlation coefficient for Freundlich isotherm is 0.994, and the Freundlich isotherm heterogeneity factor (*n*) is 3.6. For the Freundlich isotherm, ıf the parameter *n* value is between 1–10 and the 1/*n* value is between 0.1 < 1/*n* < 0.5, the process is favorable for adsorption process. In this study, the *n* value was calculated as 3.6, and the 1/*n* value was 0.27. Therefore, it was concluded that the obtained nanocomplex material is suitable for Ni(II) adsorption. [48]. It is also classified in the literature as 1/*n* = 0.1–0.5 (perfect adsorption process); 1/*n* = 0.5–1.0 (easy adsorption process); 1/*n* ≥ 1 (difficult adsorption process) [48,49] These results show that the adsorption of Nickel with GO@Fe_3_O_4_@Pluronic-F68 is physical and that a very good adsorption process is achieved at 1/*n* values (0.27). When examining the adsorption isotherms, the fact that the correlation coefficients obtained from both the Langmuir and Freundlich isotherm models are close to 1 shows that both models can be applied to this process [50].

### 3.4. Adsorption Kinetics

**Figure 11:**
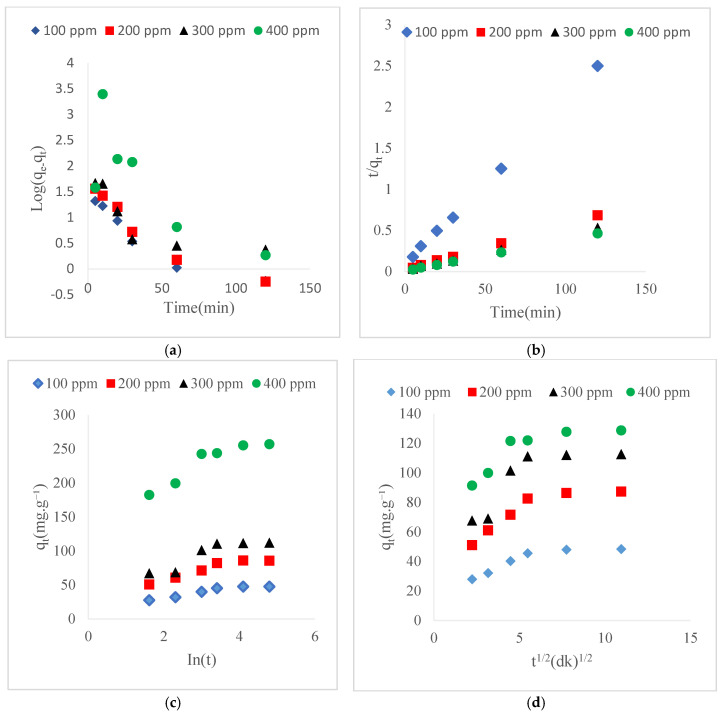
(**a**) Pseudo-first order kinetic model (**b**) Pseudo-second order kinetic model (**c**) Elovich kinetic model (**d**) Intraparticle diffusion (T = 20 °C, 185 rpm, m = 0.1 g, pH =7.0).

Kinetic studies of Ni(II) on GO@Fe_3_O_4_@Pluronic-F68 were conducted for pseudo-first order, pseudo-second order, intraparticle diffusion, and Elovich models. Linear graphs of the models are given in Figure 11, and parameter values and correlation coefficients are given in Table 2. When the R^2^ values are compared, it is seen that the correlation coefficient of the pseudo-second-order kinetic model approaches 1. Therefore, it is seen that the most suitable model, according to the pseudo-first kinetic model, is the pseudo-second-order model. The high agreement with the pseudo-second-order kinetic model shows that the adsorption rate of Ni(II) ions on the adsorbent is controlled by chemical reaction. This means that the chemisorption process occurs by the exchange or sharing of equilibrium electrons between the adsorbate and the adsorbent [51]. It is seen that the R^2^ values in the Elovich kinetic model vary between 0.79 and 0.9. This shows that chemisorption is not the only mechanism in adsorption [52]. It can be said that a heterogeneous diffusion process controlled by the reaction rate and diffusion factor takes place here. Although a mechanism is not predicted with certainty with the Elovich model, large α values showed that chemical adsorption is more dominant [53]. As shown in Figure 11 and Table 2, the C values obtained from the intraparticle diffusion model are significantly different from zero. This indicates that the linear plots do not pass through the origin, suggesting that intraparticle (pore) diffusion is not the sole rate-controlling step in the adsorption process. Therefore, other mechanisms may also contribute to the overall adsorption kinetics.

### 3.5. Adsorption Thermodynamics

**Figure 12:**
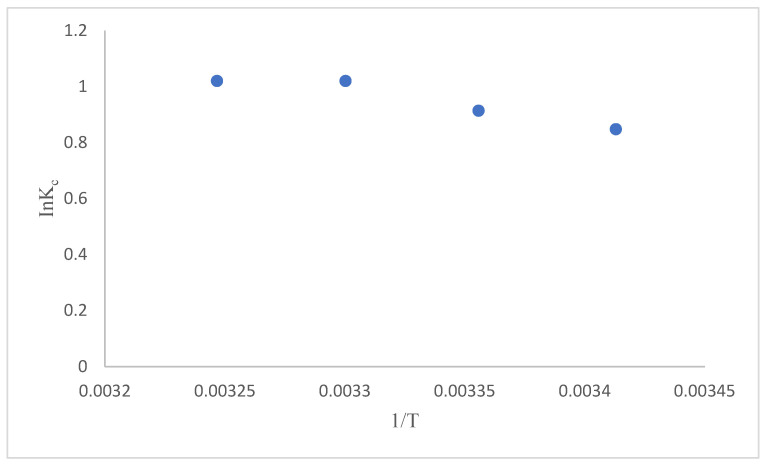
ln K_C_ − 1/T graph.

To determine the adsorption thermodynamics, pH: 7.0; 200 mg·L^−1^ Nickel solution; 4 different temperatures (20–25–30–35 °C), 0.1 g adsorbent parameters were studied. According to the experimental data using Equations (8)–(10) and Figure 12, ∆H°, ∆S°, and ∆G° parameters were calculated, and the thermodynamic parameters of the adsorption of Ni(II) onto GO@Fe_3_O_4_@Pluronic-F68 are given in Table 3.

As seen in Table 3, positive enthalpy change (ΔH° = 9.38 kJ/mol) shows that the adsorption process is endothermic. Negative ΔG° values at all temperatures show that adsorption occurs spontaneously and becomes more favorable with increasing temperature. Positive entropy change (ΔS° = 39.14) shows that the adsorption process is feasible and randomness increases at the solid-liquid interface [54].

### 3.6. Adsorption Isotherm Models: Statistical Analysis

The statistical analysis was conducted using nonlinear regression in Microsoft Excel to evaluate the adsorption equilibrium data. To elucidate the interaction mechanisms between the adsorbent surface and the adsorbate, four widely recognized adsorption isotherm models Langmuir, Freundlich, Temkin, and Dubinin-Radushkevich (D-R) were systematically assessed. The suitability of each model was determined based on the coefficient of determination (R^2^), alongside statistical validation via *p*-test and F-test values, as presented in Table 4. This comparative approach ensures robustness in identifying the model that most accurately describes the underlying adsorption phenomena.

The Langmuir isotherm model is based on the assumption that adsorption occurs on homogeneous surfaces through a monolayer mechanism and that there is no interaction between adsorption sites. In this study, the fit of the Langmuir model to experimental adsorption data was examined using statistical parameters, yielding R^2^ = 0.985, *p* < 0.001, and F = 271.46. The high R^2^ value (0.985) indicates that the model fits the experimental data to a high degree; however, the *p* value being below the significance threshold (*p* < 0.01) and the relatively low F statistic suggest that the model is statistically less reliable compared to the Freundlich isotherm. These findings suggest that the adsorption mechanism does not proceed in a completely monolayer structure and that there are deviations in surface homogeneity. Therefore, it can be proposed that factors such as multilayer adsorption or surface heterogeneity play a role in the system [19].

The Freundlich isotherm model accepts that adsorption can occur on heterogeneous surfaces through a multilayer mechanism and that intermolecular interactions exist between adsorption regions. In this study, the statistical parameters obtained for the Freundlich model (R^2^ = 0.995, *p* < 0.001, F = 582.66) demonstrated that the model showed exceptional agreement with experimental data. The model’s high R^2^ value (0.995) and statistically significant F value (582.66) are quantitative indicators that the Freundlich isotherm describes the adsorption process with the highest accuracy. Additionally, the extremely low *p* value (*p* < 0.001) confirms the model’s high statistical reliability. A comprehensive evaluation of these findings indicates that the adsorption mechanism primarily occurs as multi-layer adsorption in a system exhibiting heterogeneous surface characteristics, with adsorbate-adsorbent interactions playing a significant role [55].

The Temkin isothermal model predicts that the adsorption heat exhibits a linear distribution across the adsorbent surface and that characteristic interactions exist between the adsorbate molecules. In this study, the statistical parameters of the Temkin model (R^2^ = 0.989, *p* = 0.001, F = 262.06) were obtained, and these values revealed that the model describes the adsorption process with relatively lower accuracy compared to other isotherm models. In particular, the relatively low F statistic (262.06) and the borderline significance of the *p* value (0.001) indicate that the statistical validity of the model is limited. These findings support the idea that heat adsorption in the system exhibits a homogeneous distribution, and that the adsorption mechanism occurs under heterogeneous surface conditions. Therefore, a significant mismatch between the fundamental assumptions of the Temkin model and the experimental data has been identified [55].

The Dubinin-Radushkevich (D-R) isotherm model was developed to distinguish between physical (physisorption) and chemical (chemisorption) adsorption and plays a critical role in elucidating adsorption mechanisms, particularly for adsorbents with porous structures. In this study, the statistical performance of the D-R model was characterised by R^2^ = 0.828, *p* = 0.032, and F = 14.46. The moderate R^2^ coefficient and statistically significant *p* value (*p* < 0.05) indicate that the model shows reasonable agreement with experimental data; however, the low F statistic reveals that the model’s predictive capacity is limited. These findings suggest that physical adsorption mechanisms dominate over chemical bond formation in the system under investigation and that surface-pore structure interactions play a decisive role in the process [19].

## 4. Conclusions

In this study, we modified the GO-Fe_3_O_4_ nanocomposite with Pluronic F-68 polymer and used the resulting nanocomposite for Ni(II) removal from wastewater. Adsorbent characterization, adsorption capacity, isotherm and kinetic models, thermodynamic parameters, and comparison with other adsorbents used in Ni(II) were investigated in the adsorption process. Fourier-transform infrared spectroscopy (FTIR), scanning electron microscopy (SEM), X-ray diffraction (XRD), and Brunauer-Emmett-Teller (BET) analyses were performed for characterization. According to the correlation coefficients, the experimental data fit both the Langmuir and Freundlich isotherm models well. According to the Langmuir isotherm, the maximum adsorption capacity was calculated as 151.5 mg·g^−1^.

In the kinetic studies of the adsorption process, pseudo-first order, pseudo-second order, Elovich, and intraparticle diffusion models were evaluated, and it was observed that the second-order kinetic model had the highest correlation coefficient (R2 > 0.99) among the models. From the thermodynamic analyses, the free enthalpy change of 9.38 kJ. mol^−1^ and the Gibbs free energy value of approximately −2.06 kJ.mol^−1^ determined that the adsorption process was endothermic, physical in character, and spontaneous.

The GO-Fe_3_O_4_-PF-68 nanocomposite material presents a promising approach for the removal of heavy metals from aqueous solutions. Its synthesis, characterization, and adsorption performance are critical for evaluating its effectiveness and potential for large-scale applications. Further studies focusing on the optimization of synthesis parameters, understanding the adsorption mechanism in detail, and assessing environmental impacts are essential for the practical application of this nanocomposite material.

## Figures and Tables

**Table 1:** Parameters and correlation coefficients of the equilibrium isotherm models for the adsorption of Ni(II) onto GO@Fe_3_O_4_@Pluronic-F68 at 20 °C.

Isotherm Parameters	GO@Fe_3_O_4_@Pluronic-F68
Langmuir	
Q_m_ (mg·g^−1^)	151.5
K_L_ (L·mg^−1^)	0.06
R_L_	0.05
R^2^	0.989
Freundlich	
K_f_ [(mg·g^−1^)(L·mg^−1^)^−1/n^]	33.1
n	3.6
R^2^	0.994
Temkin	
K_T_ (L·mg^−1^)	1.59
B_T_	23.9
R^2^	0.98
Dubinin-Radushkevich	
β_DR_ (×10^−6^ mol^2^·kj^−2^)	−0.3
q_m_ (mg·g^−1^)	116.80
R^2^	0.828

**Table 2:** Parameter values and correlation coefficients of kinetic models for Ni(II) adsorption.

Kinetic Model	Parameters	Concentration (mg·L^−1^)			
		100	200	300	400
pseudo first order	k_1_	0.031	0.036	0.024	0.046
	R^2^	0.85	0.89	0.63	0.64
pseudo second order	k_2_	0.0048	0.0013	0.0011	0.016
	R^2^	0.99	0.99	0.99	0.99
Intraparticle diffusion	k_i_	2.33	4	5.3	4
	C	27.1	50.5	65.4	92.4
	R^2^	0.76	0.76	0.63	0.69
Elovich model	α	87.36	210.29	115.94	204.7
	Β	0.14	0.08	0.02	0.01
	R^2^	0.9	0.9	0.79	0.86

**Table 3:** Thermodynamic parameters of Ni(II) adsorption on GO@Fe3O4@Pluronic-F68.

T (K)	∆H° (kJ·mol^−1^)	∆S° (J·mol^−1^·K^−1^)	∆G° (kJ·mol^−1^)
293	9.38	39.14	−2.06
298			−2.26
303			−2.57
308			−2.61

**Table 4:** Isotherm models regression analysis.

Isotherm Model	R^2^	*p*-Test	F-Test
Langmuir	0.985	<0.001	271.464
Freundlich	0.995	<0.001	582.661
Temkin	0.989	0.001	262.065
Dubinin–Radushkevich	0.828	0.032	14.467

## Data Availability

The original contributions presented in this study are included in the article/Supplementary Material. Further inquiries can be directed to the corresponding authors.

## Supplementary Materials

The following supporting information can be downloaded at: https://www.mdpi.com/article/10.3390/polym17152141/s1, Figure S1: Results of FT-IR analysis after nickel adsorption; Figure S2: EDX datas after adsorption; Figure S3: Per cent weight (C, O, Fe and Ni); Figure S4: XRD datas after Ni(II) adsorption; Figure S5: SEM images of GO-Fe3O4-PF-68 nanocomposite after Ni adsorption.
