# Supplementary material for: Adsorption Capacity, Reaction Kinetics and Thermodynamic Studies on Ni(II) Removal with GO@Fe3O4@Pluronic-F68 Nanocomposite
Source: Polymers (Basel). 2025 Aug 5;17(15):2141. doi: 10.3390/polym17152141 (PMC12349223; doi:10.3390/polym17152141)
Supplement: Supplementary file 1 [file polymers-17-02141-s001.zip › polymers-3752469-supplementary.pdf]

SUPPORTING INFORMATION

Adsorption Capacity, Reaction Kinetics, and Thermodynamic Studies on Ni(II) Removal with GO@Fe_3O_4@Pluronic-F68 (GFPF-68) Nanocomposite

Ali Çiçekçi[1,2] ,Fatih Sevim[1,] , Melike Sevim[1,3], Erbil Kavcı[2]

[1]*Ataturk University, Chemical Engineering Department, Erzurum-TÜRKİYE*

[2]*Kafkas University, Chemical Engineering Department, Kars-TÜRKİYE*

[3]*Nanoscience and Nanoengineering Department, Atatürk University, 25240, Erzurum, TÜRKİYE*

* Corresponding author.

E-mail address: alicicekci3610@gmail.com, fsevim@atauni.edu.tr

Table of contents

FTIR, EDX, XRD, and SEM spectra……......…………………………………………………..2-3.

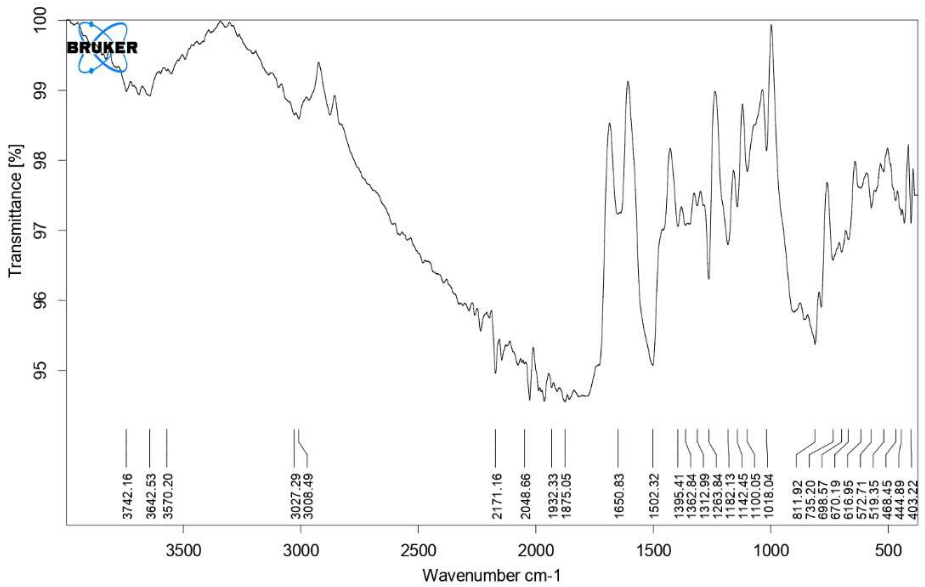

Figure S1. Results of FT-IR analysis after nickel adsorption.

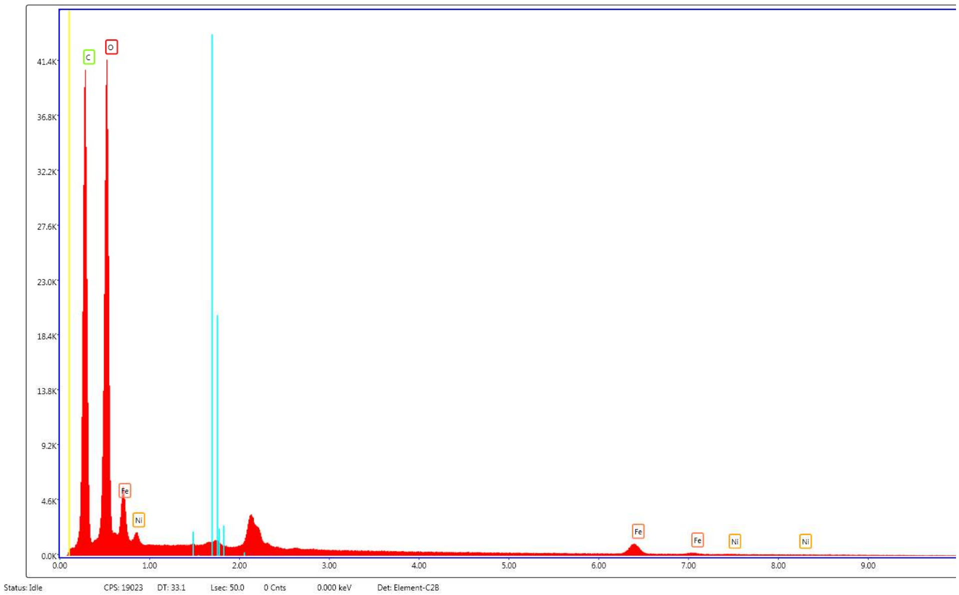

Figure S2. EDX data after adsorption.

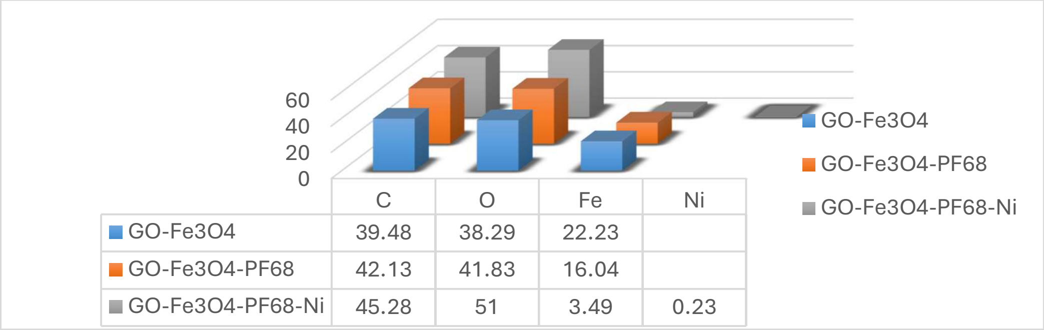

	C	O	Fe	Ni
GO-Fe3O4	39.48	38.29	22.23	
GO-Fe3O4-PF68	42.13	41.83	16.04	
GO-Fe3O4-PF68-Ni	45.28	51	3.49	0.23

Figure S3. Per cent weight (C, O, Fe, and Ni).

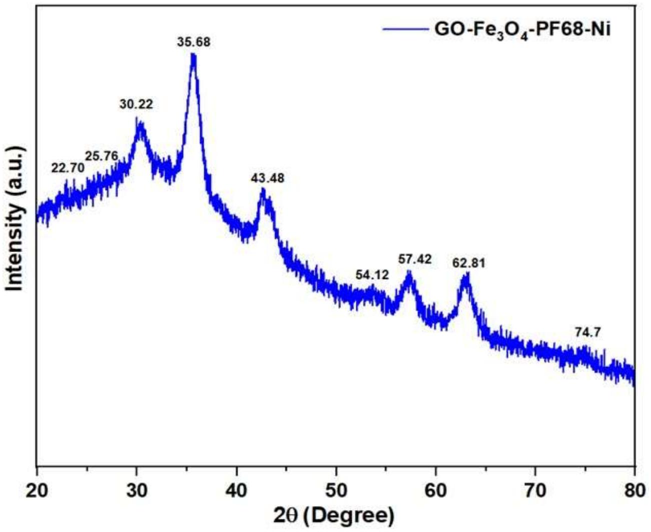

Figure S4. XRD data after Ni(II) adsorption.

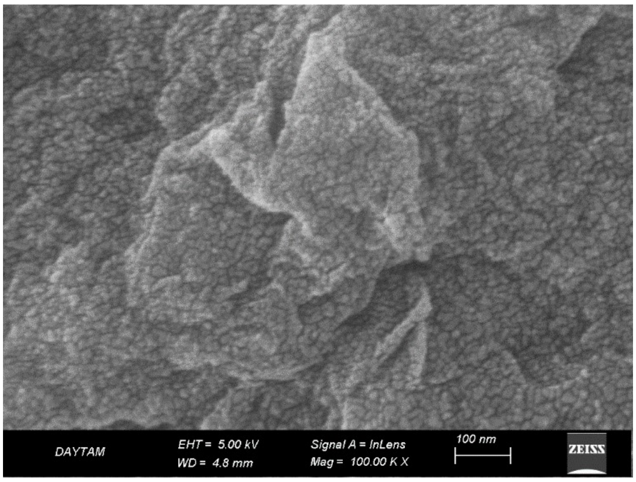

Figure S5. SEM images of GFPF-68 nanocomposite after Ni adsorption.
